# The biological carbon pump in CMIP6 models: 21st century trends and uncertainties

**DOI:** 10.1073/pnas.2204369119

**Published:** 2022-07-11

**Authors:** Jamie D. Wilson, Oliver Andrews, Anna Katavouta, Francisco de Melo Viríssimo, Ros M. Death, Markus Adloff, Chelsey A. Baker, Benedict Blackledge, Fraser W. Goldsworth, Alan T. Kennedy-Asser, Qian Liu, Katie R. Sieradzan, Emily Vosper, Rui Ying

**Affiliations:** ^a^School of Earth Sciences, University of Bristol, Bristol BS8 1RJ, United Kingdom;; ^b^School of Geographical Sciences, University of Bristol, Bristol BS8 1SS, United Kingdom;; ^c^National Oceanography Centre, Liverpool L3 5DA, United Kingdom;; ^d^Department of Earth, Ocean and Ecological Sciences, University of Liverpool, Liverpool L69 3GP, United Kingdom;; ^e^National Oceanography Centre, Southampton SO14 3ZH, United Kingdom;; ^f^Grantham Research Institute on Climate Change and the Environment, London School of Economics and Political Science, London, WC2A 3PH, United Kingdom;; ^g^School for Geography, Earth and Environmental Sciences, University of Birmingham, Birmingham B15 2TT, United Kingdom;; ^h^Department of Physics, University of Oxford, Oxford OX1 3PU, United Kingdom;; ^i^School of Ocean Sciences, Bangor University, Bangor LL57 2DG, United Kingdom

**Keywords:** carbon cycle, marine biogeochemistry, biological carbon pump, CMIP6

## Abstract

The biological carbon pump (BCP) stores ∼1,700 Pg C from the atmosphere in the ocean interior, but the magnitude and direction of future changes in carbon sequestration by the BCP are uncertain. We quantify global trends in export production, sinking organic carbon fluxes, and sequestered carbon in the latest Coupled Model Intercomparison Project Phase 6 (CMIP6) future projections, finding a consistent 19 to 48 Pg C increase in carbon sequestration over the 21st century for the SSP3-7.0 scenario, equivalent to 5 to 17% of the total increase of carbon in the ocean by 2100. This is in contrast to a global decrease in export production of –0.15 to –1.44 Pg C y^–1^. However, there is significant uncertainty in the modeled future fluxes of organic carbon to the deep ocean associated with a range of different processes resolved across models. We demonstrate that organic carbon fluxes at 1,000 m are a good predictor of long-term carbon sequestration and suggest this is an important metric of the BCP that should be prioritized in future model studies.

A fraction of the carbon fixed in the surface ocean by phytoplankton is isolated away from the atmosphere in the ocean via sinking organic detritus (particulate organic carbon [POC]) that is respired in the ocean interior, a process known as the biological carbon pump (BCP; here we focus on the organic carbon, or soft-tissue, part only). The relatively fast timescale of sinking particles (days/weeks) versus the much longer timescales of ocean circulation [10 to 1,000 y dependent on depth (1)] leads to the accumulation of ∼1,700 Pg of dissolved inorganic carbon (DIC) in the ocean beyond the concentration expected solely with physiochemical drivers, effectively lowering the baseline atmospheric CO_2_ concentration by ∼150 to 250 ppm (2). The total amount of carbon sequestered by the BCP (C^soft^) can be conceptually simplified as a function of three key processes: the flux of carbon leaving the surface ocean (export production), the average depth at which organic carbon is respired [attenuation of the POC flux with depth (3)], and how long respired DIC takes to return to the surface ocean and atmosphere [ocean residence time (1)]. All three processes are expected to change in response to a changing climate. Both export production and POC attenuation are affected by factors such as the temperature dependence of metabolic rates and the diversity of plankton communities (4), while the circulation timescales of the ocean are impacted by warming-driven stratification (5, 6) (which additionally impacts export production rates through changing nutrient fluxes). As such, the BCP is a vulnerable carbon pool in the Earth system, but there is low confidence in the magnitude and direction of this ocean carbon feedback (7).

Here we quantify trends and uncertainties in the BCP within the Coupled Model Intercomparison Project Phase 6 [CMIP6 (8)] ensemble for the historical period and two future scenarios: mitigated carbon emissions Shared Socioeconomic Pathways (SSP) (SSP1-2.6) and continued emissions (SSP3-7.0). Previous analyses of the BCP in CMIP5 models typically focused on export production as a metric of carbon storage (5, 6). We provide a broader characterization of the BCP that quantifies all processes additional to export production as well as carbon storage (*Materials and Methods*).

All models consistently predict that carbon storage by the BCP increases over the 21st century (Fig. 1 *A* and *B*); i.e., the BCP acts as sink for atmospheric CO_2_. Carbon storage relative to the preindustrial increases by 19 to 48 Pg C and by 10 to 34 Pg C for the SSP3-7.0 and SSP1-2.6 scenarios, respectively. Although consistent with previous CMIP5 estimates (9) (4 to 50 Pg C), all models substantially underestimate the increase in C^soft^ across the past 5 decades (–0.5 to 2 Pg C per decade) compared to observations (7 Pg C per decade) (10). As per CMIP5 (11), global export production at 100 m declines across the 21st century by between –0.15 and –1.44 Pg C y^–1^ with exceptions for the IPSL-CM6A-LR and IPSL-CM5A2-INCA models where the decline is much smaller (Fig. 1 *E* and *F*). Both trends reflect a common physical driver of warming-driven stratification that reduces the rate at which deep water masses return to the surface ocean (Fig. 1 *C* and *D*) (5). This acts to reduce the global supply of nutrients to the surface ocean, limiting productivity, but also the supply of respired DIC leading to the accumulation of respired CO_2_ in the ocean interior. Assuming changes in C^soft^ predict the cumulative air–sea flux of CO_2_ (12), the magnitude of change in the BCP is a small fraction (5 to 17% for both scenarios) of total carbon storage projected for the 21st century (7).

**Fig. 1.**
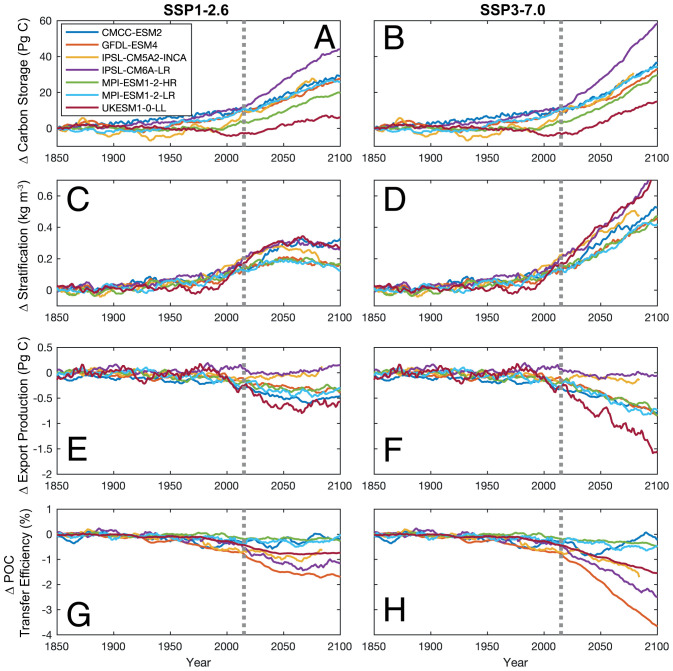
Modeled global mean historical and 21st century trends in the biological carbon pump for the SSP1-2.6 and SSP3-7.0 scenarios. (*A* and *B*) Carbon storage estimated by AOU. (*C* and *D*) Global area-weighted mean stratification index. (*E* and *F*) Export production of particulate organic carbon at 100 m. (*G* and *H*) The percentage of particulate organic carbon exported at 100 m reaching 1,000 m (transfer efficiency). *C*–*H* are smoothed with a 10-y moving average. The gray dotted lines demarcate the historical and future projections.

The biggest uncertainty in the BCP projections is the response of POC transfer efficiency to 21st century climate change with both projected increases and decreases across CMIP6 models (Fig. 1 *E* and *F*). The preindustrial global mean transfer efficiency varies widely between 3% (UKESM1-0-LL) and 25% (IPSL-CM5A2-INCA) compared with sediment trap observations of ∼20% (13). Spatial distributions of preindustrial POC transfer efficiency also vary across models, with predictions of higher transfer of export production to depth in upwelling regions (GFDL-ESM4 and MPI-ESM1-2-HR), higher transfer matching patterns of surface productivity (UKESM1-0-LL and IPSL-CM6A-LR), and higher transfer in the high latitudes (CMCC-ESM2) (Fig. 2). These differences reflect the range of processes that are variously resolved in the different biogeochemical models, including temperature and oxygen-dependent remineralization, ballasting by inorganic material, and dependence of sinking velocities on cell size and plankton groups (*SI Appendix*, *SI Methods*). The range of processes resolved by models reflects the uncertainty in the spatial patterns of transfer efficiency in observations (Fig. 2). The variability in transfer efficiency for CMCC-ESM2 (Fig. 1*F*) reflects the intensification of spatial patterns in areas such as the North Atlantic seen in Fig. 2. Notably, GFDL-ESM4 and MPI-ESM1-2-LR/HR have similar preindustrial patterns of transfer efficiency despite different underlying drivers of POC, which then deviate in their transient response under SSP3-7.0 (Fig. 2).

**Fig. 2.**
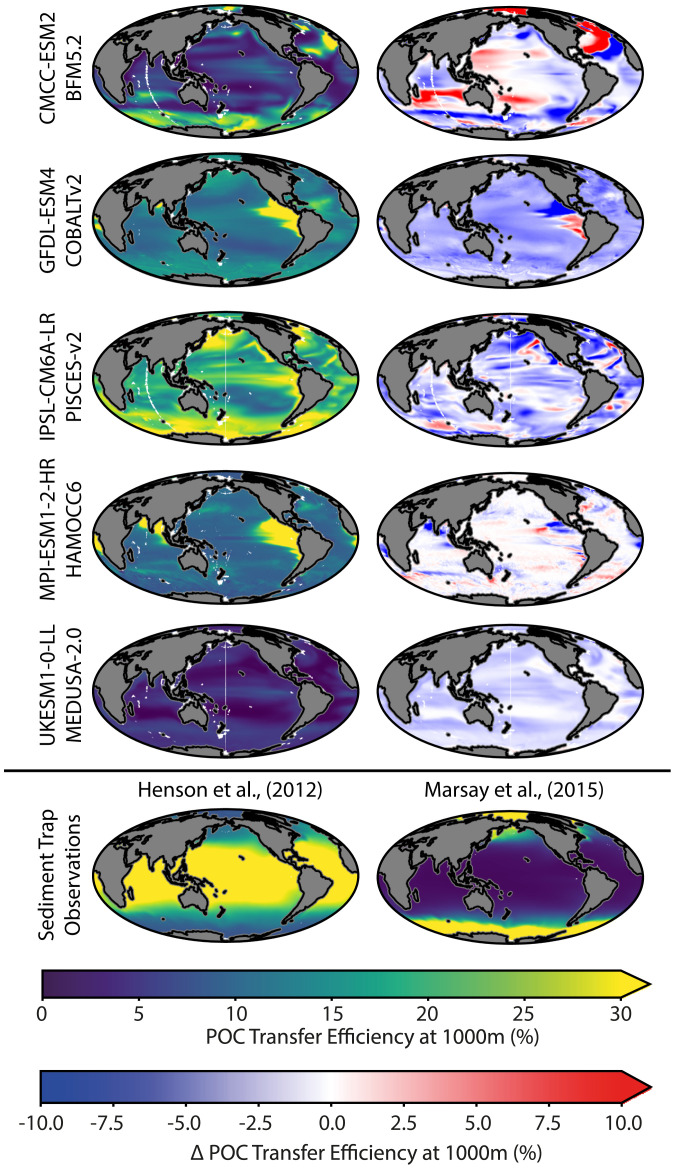
Spatial patterns in POC transfer efficiency (%) at 1,000 m for each biogeochemical model. (*Left*) The baseline preindustrial control transfer efficiency. (*Right*) The change in POC transfer efficiency between the SSP3-7.0 scenario and the preindustrial control. Spatial changes for the SSP1-2.6 scenario are similar to those of SSP3-7.0 but at a smaller magnitude. Sixth row shows transfer efficiency estimated from sediment trap observations. Model and data references are found in *SI Appendix*, *SI Methods*.

The uncertainty in POC fluxes to the deep ocean has implications for carbon storage by the BCP beyond 2100. At or near equilibrium in the preindustrial simulations, the globally integrated POC flux at 1,000 m is a better predictor of the nearly 500 Pg C difference in C^soft^ than export production across the sampled CMIP6 models (Fig. 3*B* vs. Fig. 3*A*). We further demonstrate the validity of this relationship using a first-order global mean model of the BCP (Fig. 3 *C* and *D*) (*SI Appendix*, *SI Methods*). The model predicts steady-state C^soft^ using a fixed global mean profile of ocean residence times (1) and varies export production and a POC flux curve independently within observational constraints. Assuming similarity in ocean residence times, the global mean model shows that C^soft^ could vary across models with similar export production by up to ∼1,000 Pg C, whereas it is reliably predicted by POC fluxes at 1,000 m (Fig. 3 *C* and *D*). The downward flux of POC at 1,000 m is therefore a crucial output needed in future modeling studies to assess uncertainties about the BCP and its impact on carbon sequestration. Ultimately, while the BCP is strongly determined by physical drivers in the 21st century, currently uncertain environmental and biological drivers of the BCP will have an increasing influence on carbon storage beyond 2100.

**Fig. 3.**
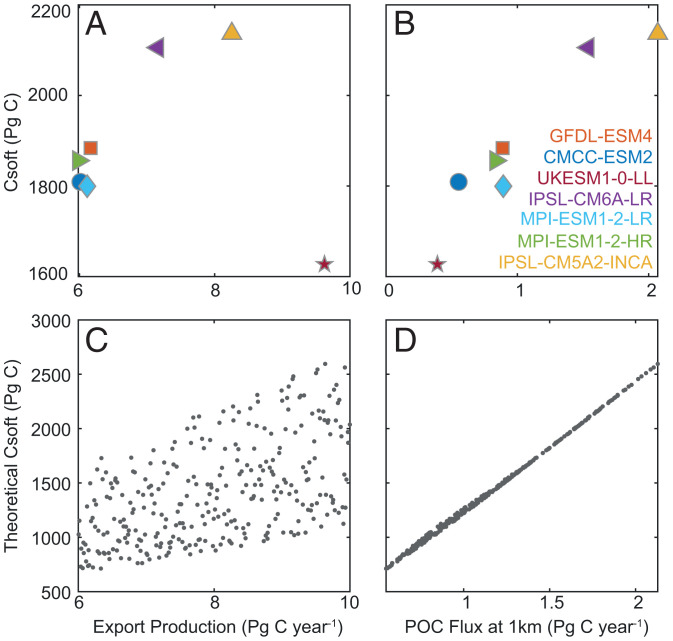
Relationship between the carbon storage by the BCP (Pg C) and (*A*) export production (Pg C y^–1^) and (*B*) downward POC flux at 1,000 m (Pg C y^–1^), for the preindustrial. Values are the mean across the control runs. The maximum relative SD across all models for each variable is <2%. (*C* and *D*) Theoretical predictions from a first-order model using a single circulation model (*SI Appendix*, *SI Methods*).

## Materials and Methods

### Particulate Organic Carbon Fluxes.

Three-dimensional fields for particulate organic carbon fluxes (*expc*) were extracted at 100 and 1,000 m where available or linearly interpolated to these depths. All POC fields were annually averaged before calculating export production.

### Apparent Oxygen Utilization and C*_soft_*.

Three-dimensional fields of apparent oxygen utilization (AOU) were calculated as

[1] $$\mathrm{AOU}=O_{2}-O_{2,\mathrm{sat}},$$

where O^2,^*^sat^* uses the oxygen solubility coefficients derived from Garcia and Gordon (14). The global mean BCP carbon storage (Pg C) was then calculated as

[2] $$C_{\mathrm{soft}}=\frac{1}{V_{\mathrm{tot}}}\int_{V}^{}[\mathrm{AOU}]R_{C:O}m_{C}dv,$$

where R_C:O_ is the stoichiometric ratio between carbon and oxygen (117:170), *m*_C_ is the molecular weight of carbon (12.01 g mol^–1^), and *V_tot_* is the total ocean volume.

### Stratification.

Stratification was calculated the area-weighted global mean difference in density at 200 m and at the surface.

### CMIP6 Models.

Fifteen CMIP6 models reported both concentrations of dissolved oxygen (*o2*) and three-dimensional fields of the downward flux of particulate organic carbon fluxes (*expc*), limited by those that reported *expc*. Of this subset, National Center for Atmospheric Research (NCAR) models did not report dissolved oxygen. Of the remaining models, eight reported output for the preindustrial, historical, and both SSP1-2.6 and SSP3-7.0 future projections experiments. We did not include MPI-ESM-1-2-HAM as it reported results up to 2050.

### Climate Signal and Drift Removal.

All output is reported as the difference to the equivalent part of the control experiment starting from the experiment branch point. The length of control run available for IPSL-CM5A-INCA limited the projection to 2080.

## Supplementary Material

Supplementary File

## Data Availability

Python code has been deposited in GitHub (DOI: 10.5281/zenodo.6481684) (15).

## References

[1] T. DeVries, F. Primeau, C. Deutsch, The sequestration efficiency of the biological pump. Geophys. Res. Lett. 39, L13601 (2012).

[2] T. Ito, M. J. Follows, Preformed phosphate, soft-tissue pump and atmospheric CO_2_. J. Mar. Res. 64, 813–839 (2005).

[3] E. Y. Kwon, F. Primeau, J. L. Sarmiento, The impact of remineralization depth on the air-sea carbon balance. Nat. Geosci. 2, 630–635 (2009).

[4] U. Passow, C. Carlson, The biological pump in a high CO_2_ world. Mar. Ecol. Prog. Ser. 470, 249–271 (2012).

[5] W. Fu, J. T. Randerson, J. K. Moore, Climate change impacts on net primary production (NPP) and export production (EP) regulated by increasing stratification and phytoplankton community structure in the CMIP5 models. Biogeosciences 13, 5151–5170 (2016).

[6] Y. Takano, I. Takamitsu, C. Deutsch, Projected centennial oxygen trends and their attribution to distinct ocean climate forcings. Global Biogeochem. Cycles 32, 1329–1349 (2018).

[7] J. Canadell ., “Global carbon and other biogeochemical cycles and feedbacks” in Climate Change 2021: The Physical Science Basis. Contribution of Working Group I to the Sixth Assessment Report of the Intergovernmental Panel on Climate Change, V. Masson-Demotte ., Eds. (Cambridge University Press, 2021), chap. 5, pp. 673–816.

[8] V. Eyring ., Overview of the Coupled Model Intercomparison Project Phase 6 (CMIP6) experimental design and organization. Geosci. Model Dev. 9, 1937–1958 (2016).

[9] A. Cabré, I. Marinov, R. Bernardello, D. Bianchi, Oxygen minimum zones in the tropical pacific across cmip5 models: Mean state differences and climate change trends. Biogeosciences 12, 5429–5454 (2015).

[10] S. Schmidtko, L. Stramma, M. Visbeck, Decline in global oceanic oxygen content during the past five decades. Nature 542, 335–339 (2017). 28202958 10.1038/nature21399

[11] L. Bopp ., Multiple stressors of ocean ecosystems in the 21st century: Projections with CMIP5 models. Biogeosciences 10, 6225–6245 (2013).

[12] W. Koeve, P. Kähler, A. Oschlies, Does export production measure transient changes of the biological carbon pump’s feedback to the atmosphere under global warming? Geophys. Res. Lett. 47, e2020GL089928 (2020).

[13] S. Henson, R. Sanders, E. Madsen, Global patterns in efficiency of particulate organic carbon export and transfer to the deep ocean. Global Biogeochem. Cycles 26, GB1028 (2012).

[14] H. E. Garcia, L. I. Gordon, Oxygen solubility in seawater: Better fitting equations. Limnol. Oceanogr. 37, 1307–1312 (1992).

[15] J. D. Wilson ., CMIP6_BCP. GitHub. https://github.com/JamieDWilson/CMIP6_BCP. Accessed 14 June 2022.

