# Supplementary material for: The biological carbon pump in CMIP6 models: 21st century trends and uncertainties
Source: Proc Natl Acad Sci U S A. 2022 Jul 11;119(29):e2204369119. doi: 10.1073/pnas.2204369119 (PMC9303979; doi:10.1073/pnas.2204369119)
Supplement: Supplementary File [file pnas.2204369119.sapp.pdf]

Supplementary Material for "Carbon Sequestration by the Biological Carbon Pump in CMIP6 models: 21st century trends and uncertainties"

Jamie D. Wilson, Oliver Andrews, Anna Katavouta, Francisco de Melo Virìssimo, Ros M. Death, Markus Adloff, Chelsey A. Baker, Benedict Blackledge, Fraser W. Goldsworth, Alan T. Kennedy-Asser, Qian Liu, Katie R. Sieradzan, Emily Vosper, Rui Ying

This supplementary material describes the methodology for the theoretical relationship between export production, downward fluxes of particulate organic carbon (POC) at 1km and carbon sequestered by the Biological Carbon Pump (C_{soft}) shown in Figure 3 of the main text. The aim is to provide theoretical support that POC fluxes at 1km are a more reliable predictor of C_{soft} given an ocean circulation at equilibrium.

S1 First Order Model of the Biological Carbon Pump

Derivation The sequestration of carbon in the ocean interior (C_{soft}) can be considered from a first-order perspective by treating the ocean interior as a simple reservoir and formulating a budget equation:

$$\frac{d\mathrm{C}_{soft}}{dt} = r - \frac{\mathrm{C}_{soft}}{\tau} \tag{1}$$

where C_{soft} is the reservoir of carbon sequestered by the biological pump (Pg C), r is the remineralisation of particulate organic carbon (POC) in the ocean interior (Pg C year^{-1}) and $\frac{\mathrm{C}_{soft}}{\tau}$ is the loss term governed by a characteristic residence time of the ocean interior (τ: year). Assuming steady-state ($\frac{d\mathrm{C}_{soft}}{dt} = 0$), we can rearrange eqn (1)

for C_{soft}:

$$C_{soft} = r\tau \tag{2}$$

We can extend equation 2 to resolve the global mean vertical variability in both remineralisation (POC fluxes that attenuate with depth[1]) and residence times which increase with depth[2].

$$C_{soft} = \sum_{z=1}^{N} r_z \tau_z \tag{3}$$

The residence times in equation 3 correspond to the first passage time: the average time for water to ventilate to the surface from a given point in the interior ocean[2] and can be readily calculated using offline circulation models[3] but is not typically applied to forward models due to a high computational cost to diagnose (see section below on Residence Times). The remineralisation of POC can be calculated from particle flux curves, such as a power-law function [1,4]. In this case, r corresponds to the vertical divergence of the particle flux curve at depth z.

Model Parameters: *Residence Times:* Global mean first passage times are diagnosed from annual-mean 2.8° MITgcm model with 15 vertical levels using an offline circulation in the form of a transport matrix[5–7]. First passage times are diagnosed using the equivalent of equation 2: by defining a constant unit flux of biogeochemically-inert

tracer to a single vertical layer in the ocean interior ($\mathbf{q}$), finding the equilibrium solution in the ocean interior ($\mathbf{c}$), and rearranging for the mean first passage time by dividing the volume-integrated inventory of $\mathbf{c}$ by the total annual flux defined in $\mathbf{q}$. In the case of linear sources/sinks, the transport matrix ($\mathbf{A}$) can be used to find the equilibrium solution for the ocean interior ($\mathbf{c}$)[6] via:

$$(\mathbf{A_i^I A_e^I} - \mathbf{I})\mathbf{c} = -\mathbf{A_i^I q} \tag{4}$$

where superscript $\mathbf{I}$ refers to the matrix for the ocean interior (all grid-boxes below the surface layer) and the subscripts $\mathbf{e}$ and $\mathbf{i}$ refer to the explicit and implicit matrices respectively. $\mathbf{I}$ is the identity matrix. The equation above is equivalent to a forward integration where a depth layer is initialised with a concentration of passive tracer, the model is integrated forward in time, and tracer that has reached the surface layer is removed. The first moment of the distribution of tracer reaching the surface over time is the mean first passage time.

POC Remineralisation Rates: We use a power-law function to describe the attenuation of sinking particulate organic carbon fluxes as a function of depth (the "Martin Curve": [1,4]):

$$F(z) = F(z_0)\left(\frac{z}{z_0}\right)^b \tag{5}$$

where F is the flux of POC at depth z, z_0 is the reference depth set to the base of the second vertical layer (120m), and b is a dimensionless exponent reflecting a linearly increasing sinking velocity with depth or decreasing remineralisation rate with depth [4]. The reference depth z_0 is typically interpreted as the export production depth. We use the inter-quartile range of exponents (b=-0.68, b=-1.13) for the power-law function, constrained by sediment trap and 234-thorium depletion observations in different regions of the ocean[8], to approximate the global-mean variability. The POC flux remaining at the bottom of the deepest depth layer is fully remineralised within that layer. We note this will lead to an over-estimation of C_{soft} as the actual global area available for remineralisation at depth is much smaller[9]. Remineralisation rates are then calculated as the vertical divergence of the POC flux curve.

Export Production We specify fixed export production values ($F(z_0)$, equation 5) defined as a characteristic range predicted by CMIP models[10] (6 to 10 Pg C year^{-1}).

Experiments: We vary export production and the Martin Curve power-law exponent within the specified ranges to generate a suite of theoretical C_{soft} values for the given global mean profile of residence times defined by the MITgcm model. We use Latin Hypercube Sampling to generate 300 stratified-random samples from uniform parameter ranges for each POC flux curve (b=-1.13 to b=-0.68) and export production (6 to 10 Pg C year^{-1}). For each of the 300 samples, the sequestered carbon (C_{soft}) is calculated according to equation 3 where r is calculated via the POC function and export production. This approach does not consider the impact of nutrient cycle feedbacks between changes

in remineralisation and export production, *e.g.,* shallower remineralisation leads to higher export production due to an increased flux of nutrients to the surface ocean[11]. As such, this approach generates a wider range of estimates than might be expected with nutrient cycling feedbacks.

S2 Biogeochemical Model Information

BFM5.2:

Sinking velocity of POC: Organic detritus has a fixed sinking velocity. Diatoms have a sinking velocity dependent on nutrient stress.

Remineralisation of POC: Function of temperature and internal nutrient quota of bacteria.

Vichi, M., Masina, S., (2007) A generalized model of pelagic biogeochemistry for the global ocean ecosystem. Part I: Theory. *Journal of Marine Systems*, 64 (1-4), 89 - 109.

COBALTv2:

Sinking velocity of POC: Fixed

Remineralisation of POC: Function of temperature, dissolved oxygen concentration and ballasting by minerals (ballasting is implemented as a fixed length-scale reflecting either faster sinking or slower remineralisation due to protection).

Stock, C.A., Dunne, J.P., Fan, S. ,Ginoux, P., ,John, J., Krasting, J.P., Laufkôtter, C.,Paulot, F., Zadeh, N. (2020) Ocean Biogeochemistry in GFDL's Earth System Model 4.1 and Its Response to Increasing Atmospheric CO_2. *Journal of Advances in Modeling Earth Systems.* 12. e2019MS002043

PISCES-v2:

Sinking velocity of POC: Small particles have a fixed sinking velocity, large particles have increasing velocity with depth. Production of small and large particles is a fixed function of calcification and silification, *i.e.,* a form of ballasting.

Remineralisation of POC: Function of temperature, dissolved oxygen concentration, aggregation dynamics.

Aumont, O., Ethé, C., Tagliabue, A., Bopp, L., Gehlen, M. (2015) PISCES-v2: an ocean biogeochemical model for carbon and ecosystem studies. *Geoscientific Model Development.* 8, 2465–2513

HAMOCC6:

Sinking velocity of POC: Fixed

Remineralisation of POC: Reduces at low dissolved oxygen concentration.

Ilyina, T., Six, K. D., Segschneider, J., Maier-Reimer, E., Li, H., and Núñez-Riboni, I. (2013), Global ocean biogeochemistry model HAMOCC: Model architecture and performance as component of the MPI-Earth system model in different CMIP5 experimental realizations. *Journal of Advances in Modeling Earth Systems*, 5, 287– 315

Mauritsen, T., Bader, J., Becker, T., Behrens, J., Bittner, M., Brokopf, R., et al. (2019). Developments in the MPI-M Earth System Model version 1.2 (MPI-ESM1.2) and its

response to increasing CO_2. *Journal of Advances in Modeling Earth Systems*, 11, 998–1038

MEDUSA-2.0:

Sinking velocity of POC: Fixed; slow and fast sinking pool

Remineralisation of POC: Temperature-dependent, zooplankton grazing (slow sinking); Ballast by minerals (fast sinking - implemented as a fixed length-scale reflecting slower remineralisation due to protection).

Yool, A., Popova, E. E., and Anderson, T. R.: MEDUSA-2.0: an intermediate complexity biogeochemical model of the marine carbon cycle for climate change and ocean acidification studies, *Geoscientific Model Development*, 6, 1767–1811

S3 Sediment Trap Observations of Transfer Efficiency

Transfer efficiencies from sediment trap observations shown in Figure 2 were derived by applying a linear fit between Martin Curve exponents (b) [1] calculated by each study with Sea Surface Temperature (deg C).

Henson et al., (2012): $b = (0.024 * SST) - 1.06$ (Stephanie Henson, pers. comm.)

Marsay et al., (2015); $b = (0.062 * SST) + 0.303$ [12]

Each linear fit was applied to climatological Sea Surface Temperature from the World Ocean Atlas 18 [13].

The transfer efficiency at 1000m (TE_{1000}) was calculated using the Martin Curve [1] with an assumed export depth of 100m:

$$TE_{1000} = \frac{1000^b}{100} \tag{6}$$

Observations from Henson et al., (2012) [14] are derived from a compilation of thorium-234 measurements and sediment traps in the deep ocean (>1000m). Observations from Marsay et al., (2015) [12] are derived from shallower (<1000m) neutrally-buoyant sediment traps.

References

1. Martin, J., Knauer, G., Karl, D. & Broenkow, W. Vertex: carbon cycling in the northeast Pacific. *Deep Sea Research* **43**, 267 – 285 (1987).

2. Primeau, F. Characterizing Transport between the Surface Mixed Layer and the Ocean Interior with a Forward and Adjoint Global Ocean Transport Model. *Journal of Physical Oceanography* **35**, 545–564 (2005). URL https://doi.org/10.1175/JPO2699.1. https://journals.ametsoc.org/jpo/article-pdf/35/4/545/4475020/jpo2699_1.pdf.

3. DeVries, T., Primeau, F. & Deutsch, C. The sequestration efficiency of the biological pump. *Geophysical Research Letters* **39** (2012). URL http://dx.doi.org/10.1029/2012GL051963. L13601.

4. Cael, B. B. & Bisson, K. Particle flux parameterizations: Quantitative and mechanistic similarities and differences. *Frontiers in Marine Science* **5**, 395 (2018). URL https://www.frontiersin.org/article/10.3389/fmars.2018.00395.

5. Khatiwala, S., Visbeck, M. & Cane, M. A. Accelerated simulation of passive tracers in ocean circulation models. *Ocean Modelling* **9**, 51 – 69 (2005). URL http://www.sciencedirect.com/science/article/pii/S1463500304000307.

6. Khatiwala, S. A computational framework for simulation of biogeochemical tracers in the ocean. *Global Biogeochemical Cycles* **21**, GB3001 (2007). URL http://dx.

doi.org/10.1029/2007GB002923.

7. Wilson, J. D., Barker, S., Edwards, N. R., Holden, P. B. & Ridgwell, A. Sensitivity of atmospheric co_2 to regional variability in particulate organic matter remineralization depths. *Biogeosciences* **16**, 2923–2936 (2019). URL https://www.biogeosciences.net/16/2923/2019/.

8. Gloege, L., McKinley, G. A., Mouw, C. B. & Ciochetto, A. B. Global evaluation of particulate organic carbon flux parameterizations and implications for atmospheric pco2. *Global Biogeochemical Cycles* **31**, 1192–1215 (2017). URL https://agupubs.onlinelibrary.wiley.com/doi/abs/10.1002/2016GB005535. https://agupubs.onlinelibrary.wiley.com/doi/pdf/10.1002/2016GB005535.

9. Menard, H. W. & Smith, S. M. Hypsometry of ocean basin provinces. *Journal of Geophysical Research* **71**, 4305–4325 (1966). URL http://dx.doi.org/10.1029/JZ071i018p04305.

10. Séférian, R. *et al.* Tracking improvement in simulated marine biogeochemistry between CMIP5 and CMIp6. *Current climate change reports* 1–25 (2020). URL https://pubmed.ncbi.nlm.nih.gov/32837849.

11. Kwon, E. Y., Primeau, F. & Sarmiento, J. L. The impact of remineralization depth on the air-sea carbon balance. *Nature Geoscience* **2**, 630–635 (2009). URL http://dx.doi.org/10.1038/ngeo612.

12. Marsay, C. *et al.* Attenuation of sinking particulate organic carbon flux through the mesopelagic ocean. *Proceedings of the National Academy of Sciences* **112**, 1089–1094 (2015). URL `http://www.pnas.org/content/112/4/1089.abstract`.

13. Locarnini, A. V. M. O. K. B. T. P. B. M. M. Z. H. E. G. J. R. R. D. S. K. W. C. R. P., R. A. & Smolyar, I. World ocean atlas 2018, volume 1: Temperature. In Mishonov, A. (ed.) *NOAA Atlas NESDIS 81*, 52 (2018).

14. Henson, S., Sanders, R. & Madsen, E. Global patterns in efficiency of particulate organic carbon export and transfer to the deep ocean. *Global Biogeochemical Cycles* **26**, GB1028 (2012).
